# Supplementary material for: RNA length has a non-trivial effect in the stability of biomolecular condensates formed by RNA-binding proteins
Source: PLoS Comput Biol. 2022 Feb 2;18(2):e1009810. doi: 10.1371/journal.pcbi.1009810 (PMC8896709; doi:10.1371/journal.pcbi.1009810)
Supplement: S1 Text — Mpipi model, full-FUS sequence, patchy particle protein/RNA model, Direct Coexistence calculations for extracting phase diagrams, and calculation of protein/RNA molecular contacts. Table A. System sizes and simulation details. Summary of the simulation details of the employed systems: Total number of proteins (NP), total number of RNA nucleotides (or RNA beads in the minimal model; NN), total number of RNA chains (NRNA,chain), length of the RNA chains (LRNA), net charge of the system, box dimensions (in x/Å, y/Å, z/Å), and estimated critical temperature (Tc in K for the high-resolution Mpipi model and in reduced units for the minimal CG model). Fig A. Direct Coexistence simulation of PR25 in absence of RNA. Snapshot of a pure PR25 Direct coexistence simulation at T/Tc,FUS = 0.5. As it can be seen, in absence of RNA, PR25 cannot undergo LLPS (even at low temperatures). The same colour code employed in Fig 1 of the main text has been employed here. Fig B. Direct Coexistence simulation of cognate proteins in absence of RNA. Snapshot of a pure cognate system Direct Coexistence simulation at T/Tc,Scaffold = 0.6. As it can be seen, in absence of RNA, the cognate protein cannot undergo LLPS (even at low temperatures). The same colour code employed in Fig 1 of the main text has been employed here. Fig C. Radius of gyration of FUS and PR25. Radius of gyration (Rg) distribution function for: a) FUS within a FUS-polyU(400-nt) condensate (green curve) and FUS in the dilute phase (black curve) at T/Tc,FUS = 0.96. b) PR25 within a PR25-polyU(400-nt) condensate (green curve) and PR25 in the dilute phase (black curve) at T/Tc,FUS = 0.85. The polyU/FUS mass ratio was kept constant at a value of 0.096, while the polyU/PR25 mass ratio at a value of 1.20. Fig D. Phase diagrams of FUS-polyU and PR25-polyU mixtures using the HPS and KH models. Temperature–density phase diagrams of FUS with polyU of different lengths at a constant polyU/FUS mass ratio of 0.16, and for a pure system of FUS [file pcbi.1009810.s001.pdf]

Supporting Information: RNA length has a non-trivial effect in the stability of biomolecular condensates formed by RNA-binding proteins

Ignacio Sanchez-Burgos[1‡], Jorge R. Espinosa[1‡], Jerelle A. Joseph[1,2,3], Rosana Collepardo-Guevara[1,2,3,*],

1 Maxwell Centre, Cavendish Laboratory, Department of Physics, University of Cambridge, J J Thomson Avenue, Cambridge, United Kingdom
2 Department of Chemistry, University of Cambridge, Lensfield Road, Cambridge, United Kingdom
3 Department of Genetics, University of Cambridge, Downing Site, Cambridge, United Kingdom

‡These authors contributed equally to this work.
* rc597@cam.ac.uk

Models and simulation details

Mpipi Model

For our simulations of FUS (see sequence below) and PR_{25} with poly-U, we employ the high-resolution sequence-dependent coarse-grained (CG) model Mpipi [1], which describes almost quantitatively the temperature-dependent LLPS phase behaviour of different protein condensates such as that of fused in sarcoma (FUS). In addition, this model correctly predicts the multiphase behaviour of the PolyR/PolyK/PolyU system, and recapitulates experimental LLPS trends for sequence mutations on FUS, DDX4 NTD and LAF-1 RRG domain variants [1]. Within this force field, electrostatic interactions are modelled with a Coulomb term with Debye–Huckel electrostatic screening [2], given by the sum over all particle-particle (i,j) interactions as:

$$E_{elec} = \sum_{i,j} \frac{q_i q_j}{4\pi\epsilon_r\epsilon_0 r_{ij}} exp(-\kappa r_{ij}) \tag{1}$$

where q is the charge (being -0.75e for the different nucleotides: A, C, G, U; and +0.75e for amino acids such as R and K, +0.375e for H, and -0.75e for D and E residues), $\epsilon_r = 80$ is the relative dielectric constant of water, ϵ_0 is the electric constant, $\kappa^{-1} = 795$ pm is the Debye screening length, and r_{ij} is the distance separating particles i and j. For these interactions, a Coulomb cut-off of 3.5 nm is employed. The non-bonded interactions between protein/RNA beads are modelled via the Wang–Frenkel potential [3]

$$E_{WF} = \sum_{i,j} \epsilon_{ij}\alpha_{ij} \left[\left(\frac{\sigma_{ij}}{r_{ij}}\right)^{2\mu_{ij}} - 1\right] \left[\left(\frac{\sigma_{ij}}{r_{ij}}\right)^{2\mu_{ij}} - 1\right]^{2\nu_{ij}} \tag{2}$$

where

$$\alpha_{ij} = 2\nu_{ij} \left(\frac{R_{ij}}{\sigma_{ij}}\right)^{2\mu_{ij}} \left[\frac{2\nu_{ij}+1}{2\nu_{ij}\left[\left(\frac{R_{ij}}{\sigma_{ij}}\right)^{2\mu_{ij}} - 1\right]}\right]^{2\mu_{ij}+1} \tag{3}$$

representing σ the molecular diameter of each residue/nucleotide and ϵ the interaction strength between distinct amino acids and nucleotides (i and j). μ_{ij} and R_{ij} are constant model parameters set to $\mu_{ij} = 1$ and $R_{ij} = 3\sigma_{ij}$ for every interaction, while σ_{ij} and ϵ_{ij} are specified for each pair of interaction in Ref. [1]. Finally, bond energy is computed with an harmonic bond potential of the following form:

$$E_{bond} = \sum_{b} \frac{1}{2} k(r_b - r_0) \quad (4)$$

where b is the total number of bonds, r_b is the bond distance, k=8.03 Jmol^{-1}pm^{-2} is the spring constant and r_0 is the bond reference position, set to 381 pm and 500 pm for protein and RNA bonds respectively. For further details on this force field and the full list of the model parameters please see Ref. [1].

FUS Sequence and PDB of the structured domains

Full-FUS sequence

MASNDYTQQATQSYGAYPTQPGQGYSQQSSQPYGQQSYSGYSQSTDTSGYGQSSYSSYGQ
SQNTGYGTQSTPQGYGSTGGYGSSQSSQSSYGQQSSYPGYGQQPAPSSTSGSYGSSSQSSSYGQ
PQSGSYSQQPSYGGQQQSYGQQQSYNPPQGYGQQNQYNSSSGGGGGGGGGGNYGQDQSSMS
SGGGSGGGYGNQDQSGGGGSGGYGQQDRGGRGRGGSGGGGGGGGGGYNRSSGGYEPRGRG
GGRGGRGGMGGSDRGGFNKFGGPRDQGSRHDSEQDNSDNNTIFVQGLGENVTIESVADYFKQ
IGIIKTNKKTGQPMINLYTDRETGKLKGEATVSFDDPPSAKAAIDWFDGKEFSGNPIKVSFATRR
ADFNRGGGNGRGGRGRGGPMGRGGYGGGGSGGGGRGGFPSGGGGGGGQQRAGDWKCPNP
TCENMNFSWRNECNQCKAPKPDGPGGGPGGSHMGGNYGDDRRGGRGGYDRGGYRGRGGDRG
GFRGGRGGGDRGGFGPGKMDSRGEHRQDRRERPY

The Uniprot code of the sequence is K7DPS7. The following Protein Data Bank (PDB) codes were used to build the globular structured domains of FUS (residues from 285–371 (PDB code: 2LCW) and from 422–453 (PDB code: 6G99)).

Minimal protein/RNA model

For the minimal coarse-grained simulations, we employ a patchy particle model [4–7] in which proteins are described by a pseudo hard-sphere (PHS) potential [8] that accounts for their excluded volume:

$$E_{PHS} = \sum_{i<j} \begin{cases} \lambda_r (\frac{\lambda_r}{\lambda_a})^{\lambda_a} \varepsilon_R \left[(\frac{\sigma}{r_{ij}})^{\lambda_r} - (\frac{\sigma}{r_{ij}})^{\lambda_a} \right] + \varepsilon_R; & \text{if } r < (\frac{\lambda_r}{\lambda_a})\sigma \\ 0; & \text{if } r \geq (\frac{\lambda_r}{\lambda_a})\sigma \end{cases}. \quad (5)$$

where λ_a =49 and λ_r=50 are the exponents of the attractive and repulsive terms respectively, and ε_R accounts for the energy shift of the pseudo hard-sphere. On top of this, we add a continuous square-well (CSW) potential for modeling the different protein binding sites, therefore mimicking protein multivalency:

$$E_{CSW} = \sum_{i<j} -\frac{1}{2} \epsilon_{CSW} \left[1 - tanh(\frac{r_{ij} - r_w}{\alpha}) \right] \quad (6)$$

where ϵ_{CSW} is the depth of the potential energy well, r_w the radius of the attractive well, and α controls the steepness of the well. We choose $\alpha = 0.005\sigma$ and $r_w = 0.12\sigma$ so that each binding site can only interact with another single one. RNA-protein interactions are modeled with a standard Lennard-Jones (LJ) potential [9]:

$$E_{LJ} = \sum_{i<j} 4\epsilon_{LJ} \left[\left(\frac{\sigma}{r_{ij}} \right)^{12} - \left(\frac{\sigma}{r_{ij}} \right)^{6} \right] \quad (7)$$

where ϵ_{LJ} measures the depth of potential and σ the excluded volume between proteins and RNA. The Lennard-Jones potential is employed between proteins and RNA beads, while RNA-RNA interactions

are modelled via the PHS potential [8]. In this way, we model RNA as a self-repulsive polymer of bonded hard spheres, and protein-RNA interactions via sites that are not the protein-protein binding sites, hence, if one protein is bonded to RNA, it can still bind to other proteins. The mass of each patch is a 5% of the central PHS particle mass, which is set to 3.32x10^{-26}kg, despite being this choice irrelevant for equilibrium simulations. This 5% ratio fixes the moment of inertia of the patchy particles (our minimal proteins). The molecular diameter of the proteins, both scaffold and cognate proteins, as well as the RNA beads is $\sigma = 0.3405$ nm, and the value of ε_R/k_B is 119.81K. With this model, we express magnitudes in reduced units: reduced temperature is defined as $T^* = k_BT/\epsilon_{CSW}$, reduced density as $\rho^* = (N/V)\sigma^3$, reduced pressure as $p^* = p\sigma^3/(k_BT)$, and reduced time as $\sqrt{\sigma^2 m/(k_BT)}$. In order to keep the PHS interaction as similar as possible to a pure HS interaction, we fix k_BT/ε_R at a value of 1.5 as suggested in Ref. [8] (fixing $T = 179.71$K). We then control the effective strength of the binding protein attraction by varying ϵ_{CSW} such that the reduced temperature, $T^* = k_BT/\epsilon_{CSW}$, is of the order of $\mathcal{O}(0.1)$. The cut-off distance for the interactions in this model are 1.17σ for the PHS and CSW potentials and 5σ for the LJ interactions. The ϵ_{LJ}/k_B for LJ interactions is set to 152.5K.

This model has been proven to qualitatively reproduce the effect of protein valency in LLPS [5], the enhancement of RNA-mediated LLPS with RNA-binding proteins [10] or the size conservation of condensates through interfacial free energy reduction [7].

Simulation details

Our Direct Coexistence simulations are performed in the NVT ensemble (i.e. constant number of particles (N), volume (V) and temperature (T)), for which we use a Nosé–Hoover thermostat [11, 12] with a relaxation time of 5 ps for the Mpipi model simulations and 0.074 in reduced units for the patchy particle simulations. Since all our potentials are continuous and differentiable, we perform all our simulations using the LAMMPS Molecular Dynamics package [13]. Periodic boundary conditions are used in the three directions of space. The timestep chosen for the Verlet integration of the equations of motion is 10 fs for the Mpipi model and $3.7x10^{-4}$ in reduced time units for the patchy particle model. In Table A, we summarise all the simulation details regarding the employed systems for both resolution models.

Computing phase diagrams via Direct Coexistence

To calculate the coexisting densities of the phase diagrams, we employ the Direct Coexistence method [14–16]. Within this scheme, the two coexisting phases are simulated by preparing periodically extended slabs of the two phases, the condensed and the diluted one, in the same simulation box. We use an implicit solvent model; accordingly, the diluted phase (protein-poor liquid phase) and the condensed phase (protein-rich liquid phase) are effectively a vapour and a liquid phase, respectively. Once our DC simulations have reached equilibrium, we compute the density profile along the long axis of the box, and thus, we extract the density of the two coexisting phases (as shown in the Supporting Material of Ref. [10]). From the plateau of the condensed phase and the diluted one, we measure the density (avoiding the interfaces between both phases). To estimate the critical point of the phase diagrams, we use the universal scaling law of coexistence densities near a critical point [17], and the law of rectilinear diameters [18]:

$$\big(\rho_l(T) - \rho_v(T)\big)^{3.06} = d\bigg(1 - \frac{T}{T_c}\bigg) \tag{8}$$

and

$$(\rho_l(T) + \rho_v(T))/2 = \rho_c + s_2(T_c - T) \tag{9}$$

where ρ_l and ρ_v refer to the coexisting densities of the condensed and diluted phases respectively, while ρ_c is the critical density, T_c is the critical temperature, and d and s_2 are fitting parameters.

System	N_P	N_N	$N_{RNA,chain}$	L_{RNA}	Net charge	Box dimensions	T_c/K (or T_c^*)
FUS+polyU	48	800	1	800	-42e	210 x 210 x 860	367
FUS+polyU	48	800	2	400	-42e	210 x 210 x 860	365.5
FUS+polyU	48	800	4	200	-42e	210 x 210 x 860	364
FUS+polyU	48	800	8	100	-42e	210 x 210 x 860	364.5
FUS+polyU	48	800	16	50	-42e	210 x 210 x 860	365
FUS+polyU	48	800	32	25	-42e	210 x 210 x 860	363
PR_{25}+polyU	98	2400	3	800	0	150 x 150 x 560	432
PR_{25}+polyU	98	2400	6	400	0	150 x 150 x 560	360
PR_{25}+polyU	98	2400	30	80	0	150 x 150 x 560	337
PR_{25}+polyU	98	2400	60	40	0	150 x 150 x 560	330
PR_{25}+polyU	98	2400	120	20	0	150 x 150 x 560	307
Scaffold+RNA	1000	250	1	250	-	30 x 30 x 270	0.14
Scaffold+RNA	1000	250	2	125	-	30 x 30 x 270	0.137
Scaffold+RNA	1000	250	5	50	-	30 x 30 x 270	0.139
Scaffold+RNA	1000	250	10	25	-	30 x 30 x 270	0.136
Scaffold+RNA	1000	250	25	10	-	30 x 30 x 270	0.131
Cognate+RNA	1000	250	1	250	-	33 x 33 x 250	0.121
Cognate+RNA	1000	250	2	125	-	33 x 33 x 250	0.125
Cognate+RNA	1000	250	5	50	-	33 x 33 x 250	0.122
Cognate+RNA	1000	250	10	25	-	33 x 33 x 250	0.118
Cognate+RNA	1000	250	25	10	-	33 x 33 x 250	0.097
Cognate+RNA	1000	250	50	5	-	33 x 33 x 250	0.081

Table A. Summary of the simulation details of the employed systems: Total number of proteins (N_P), total number of RNA nucleotides (or RNA beads in the minimal model; N_N), total number of RNA chains ($N_{RNA,chain}$), length of the RNA chains (L_{RNA}), net charge of the system, box dimensions (in x/Å, y/Å, z/Å), and estimated critical temperature (T_c in K for the high-resolution Mpipi model and in reduced units for the minimal CG model).

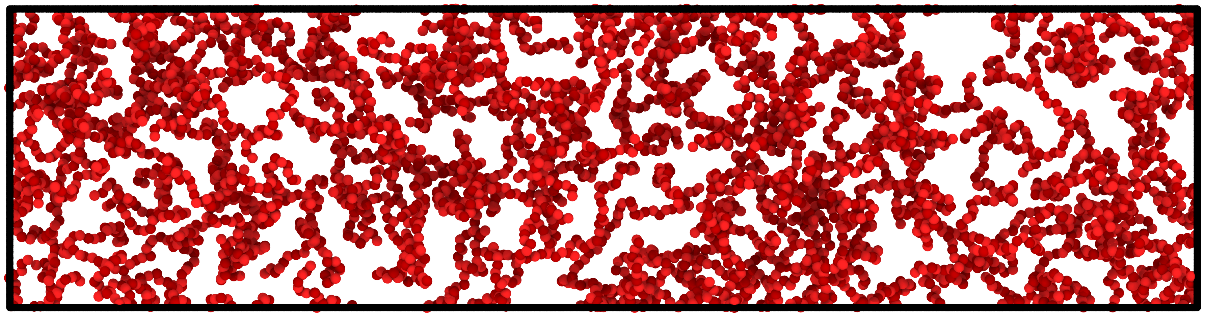

Fig A. Snapshot of a pure PR_{25} Direct coexistence simulation at $T/T_{c,FUS}$=0.5. As it can be seen, in absence of RNA, PR_{25} cannot undergo LLPS (even at low temperatures). The same colour code employed in Fig. 1 of the main text has been employed here.

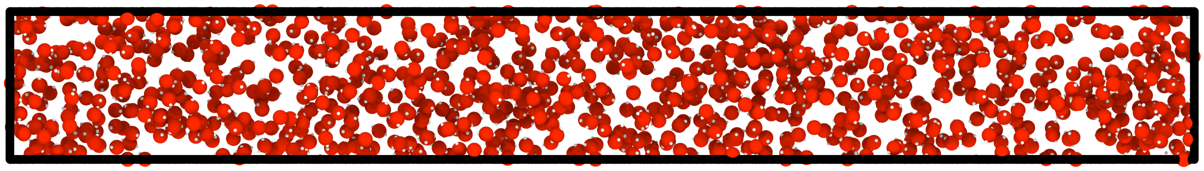

Fig B. Snapshot of a pure cognate system Direct Coexistence simulation at $T/T_{c,Scaffold}$=0.6. As it can be seen, in absence of RNA, the cognate protein cannot undergo LLPS (even at low temperatures). The same colour code employed in Fig. 1 of the main text has been employed here.

Results with the HPS & KH models for FUS and PR_{25}

Additionally to the results presented in the main text of this article, we perform simulations of FUS and PR_{25} in presence of RNA with different lengths at a constant RNA/protein concentration employing the hydrophobicity scale (HPS) model for the FUS-polyU systems, and the Kim-Hummer (KH) model for those of PR_{25} with polyU. Both models are detailed in Ref. [19]. In these models, the solvent is also implicit as in the Mpipi force field [1]. The simulation details are the same provided for the Mpipi simulations in Section 1D. Here, polyU RNA strands are mimicked as chains of glutamic acid (and so its mass) as shown in Ref. [20]. In Fig D (a) we show how the phase behaviour of FUS and polyU RNA deeply resembles the one observed in the main document using the Mpipi model (Fig 3 (a) of the main text): the critical temperature of FUS-RNA mixtures marginally depends on the length of the added polyU strands. However, for PR_{25}-polyU systems (Fig D (b)), there is a remarkable difference of a factor of 2 (almost 200K) between the shortest (20-nucleotide long RNA chains) and the longest ones (800-nucleotide long RNA chains) as observed in the main text for the Mpipi model (Fig 3 (b) of the main text).

Moreover, the same intermolecular contact analysis of Fig 4 of the main text was also performed for these simulations, providing similar results to those computed from the Mpipi model calculations. In Fig E(a), it can be seen how FUS-FUS contacts are mostly responsible of holding the protein condensates (displaying about 4 times more contacts per nm^3 than the RNA-FUS contacts), while in the PR_{25}-RNA condensates, heterotypic PR_{25}-RNA interactions are predominant respect to those of PR_{25}-PR_{25} (Fig E (b)). Lastly, the length dependent behaviour of the critical temperature for PR_{25} condensates is held as in the case of the Mpipi model (main document, Fig 4(d)), while for FUS-poly-U systems the critical temperature remains roughly constant independently of the RNA chain length.

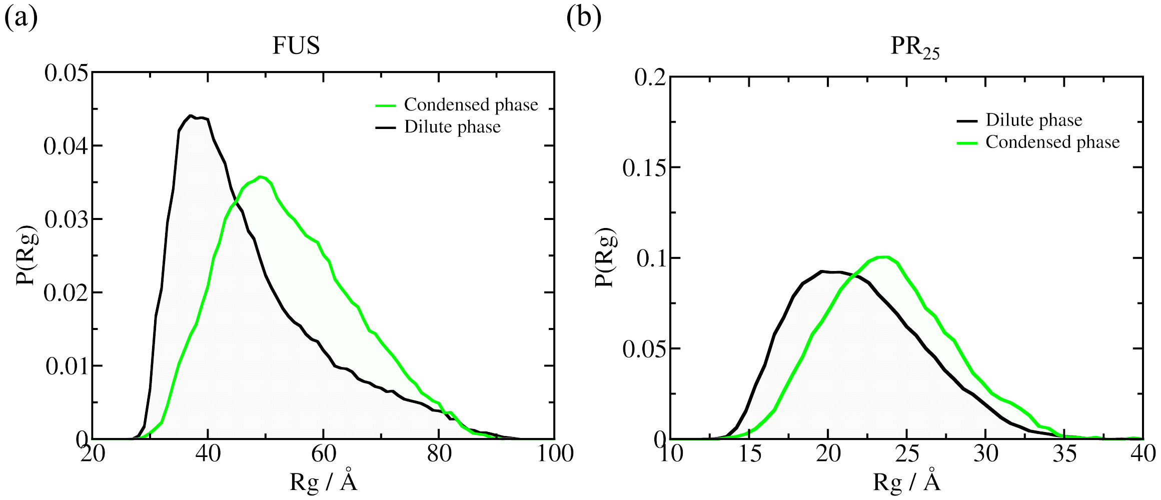

Fig C. Radius of gyration (R_g) distribution function for: a) FUS within a FUS-polyU(400-nt) condensate (green curve) and FUS in the dilute phase (black curve) at $T/T_{c,FUS}$=0.96. b) PR_{25} within a PR_{25}-polyU(400-nt) condensate (green curve) and PR_{25} in the dilute phase (black curve) at $T/T_{c,FUS}$=0.85. The polyU/FUS mass ratio is kept constant at a value of 0.096, while the polyU/PR_{25} mass ratio at a value of 1.20.

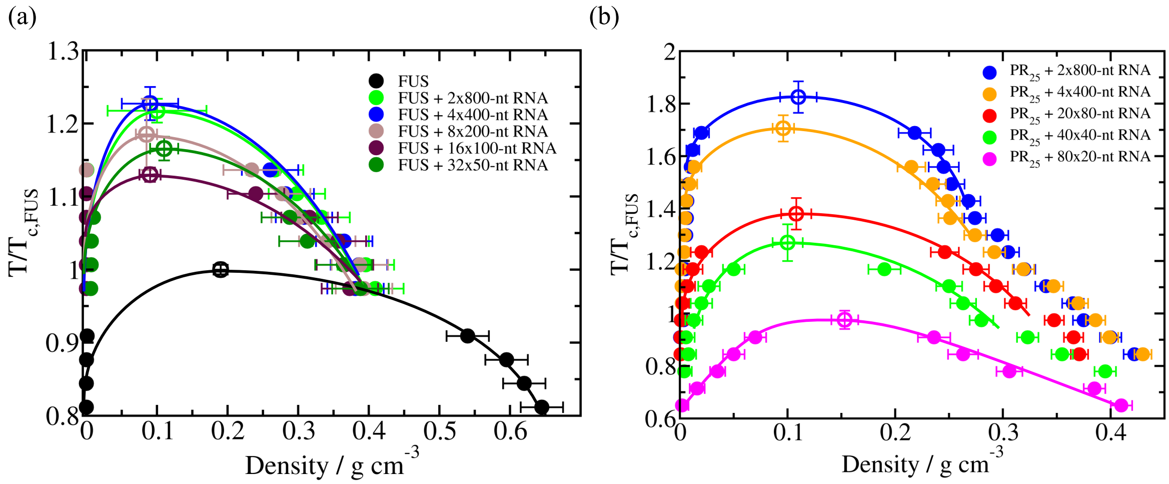

Fig D. Temperature–density phase diagrams of FUS with polyU of different lengths at a constant polyU/FUS mass ratio of 0.16, and for a pure system of FUS (black curve). (b) Temperature–density phase diagrams of PR_{25} with RNA at different lengths at a constant RNA/PR_{25} mass ratio of 0.57. In both (a) and (b) panels, filled circles represent the coexisting densities evaluated from DC simulations while empty circles depict the critical temperatures estimated from the law of rectilinear diameters and critical exponents [17] near the critical temperature. Temperature in both panels has been normalized by the critical temperature of pure FUS, $T_{c,FUS}$=309K (black empty circle in (a)).

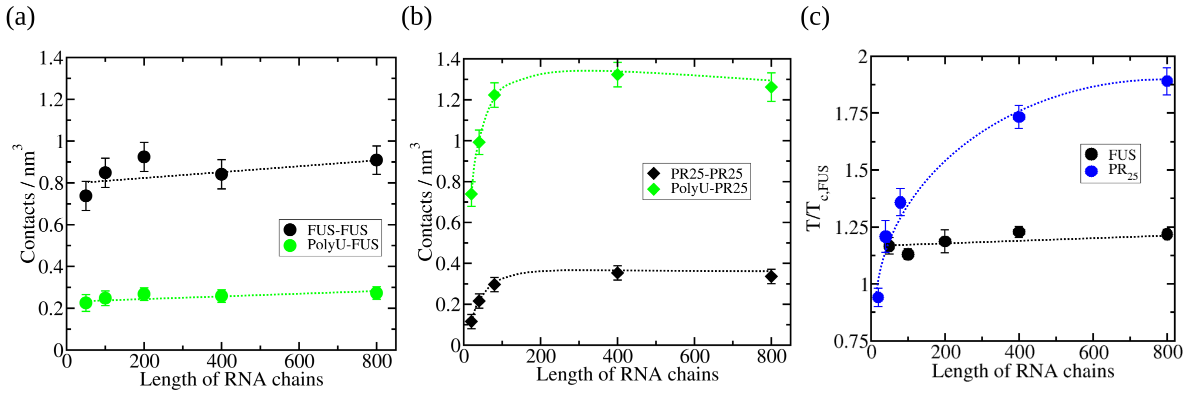

Fig E. Density of LLPS-stabilizing intermolecular contacts within condensates as a function of RNA length plotted separately for protein–protein interactions (black symbols) and protein–RNA interactions (green symbols) for FUS-polyU (a) and PR_{25}-polyU mixtures (b). The temperature at which the intermolecular contacts were computed was $T/T_{\mathrm{c,FUS}}$=1.13 for FUS–RNA systems and $T/T_{\mathrm{c,FUS}}$=0.924 for PR_{25}–RNA mixtures (the highest temperature at which all systems with distinct RNA lengths can phase separate). (c) Critical temperature *versus* RNA length for FUS–RNA (black) and PR_{25}–RNA (blue) systems.

Computing the number of intermolecular contacts

In Figs 4 and 6 of the main document, we provide the number of intermolecular contacts per unit of volume. To compute such magnitude, we consider two amino acids to be in contact under a distance of 7.7Å, which is the average of all the possible σ_{ij} (Eq. 2) multiplied by a factor 1.2 (typical value in σ at which the attractive LJ/Wang-Frenkel interactions become mild). The RNA-protein interactions are considered as contacts when the distance between a nucleotide and an amino acid is lower than 8.95Å, which is the average σ_{ij} between uridine and any other amino acid, multiplied also by a factor of 1.2. The number of total contacts is averaged throughout entire converged simulations of about 2 μs. Then, the contacts density is obtained by dividing the total number of contacts within the condensed phase by the condensate volume. The density of LLPS-stabilizing intermolecular contacts within the condensates as a function of temperature for FUS+polyU(400-nt) and PR_{25}+polyU(400-nt) is provided in Fig. F (a) and (b) respectively. Moreover, the electrostatic *vs.* non-electrostatic contribution to the attractive potential interactions sustaining the phase-separated condensates as a function of RNA length for FUS+polyU condensates (filled circles) and PR_{25}+polyU condensates (empty squares) is given in Fig. G.

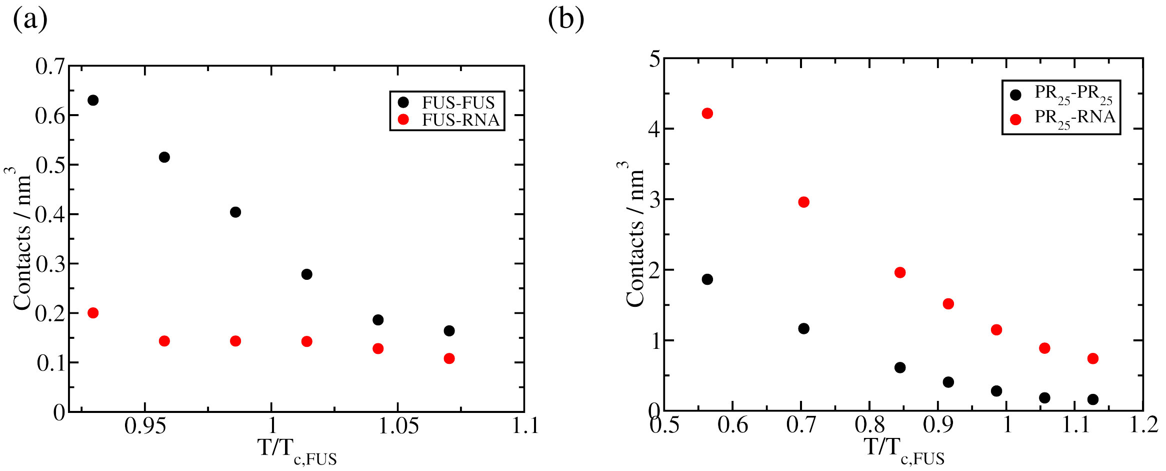

Fig F. Density of LLPS-stabilizing intermolecular contacts within the condensates (plotted separately for protein–protein interactions, black circles, and protein–RNA interactions, red circles) as a function of temperature (T/T$_{c,FUS}$) for: a) FUS+polyU(400-nt), and b) PR$_{25}$+polyU(400-nt) condensates. The polyU/FUS mass ratio was kept constant at a value of 0.096 for all FUS simulations, while the polyU/PR$_{25}$ mass ratio at a value of 1.20 for all PR$_{25}$ simulations at every studied temperature.

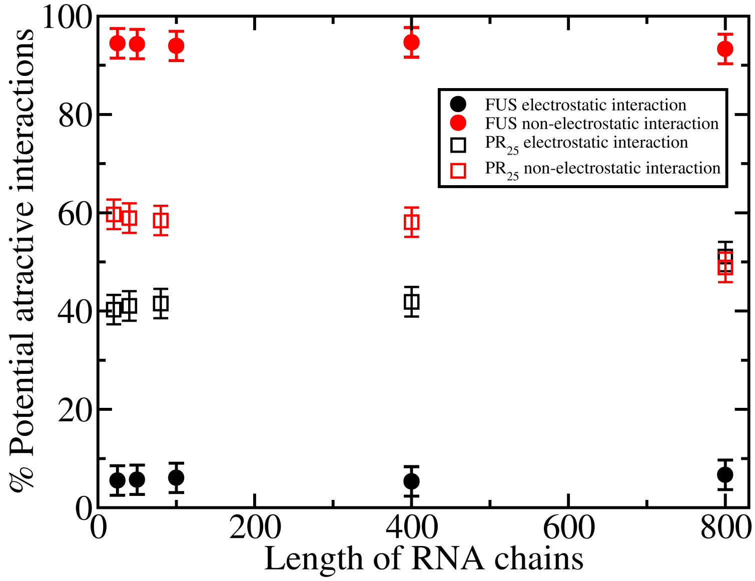

Fig G. Electrostatic (black symbols) *vs.* non-electrostatic (red symbols) contribution to the potential attractive interactions (molecular contacts sustaining phase-separated condensates) as a function of RNA length for FUS+polyU condensates (filled circles) and PR$_{25}$+polyU condensates (empty squares). The temperature at which the intermolecular contacts were computed was T/T$_{\mathrm{c,FUS}}$=0.99 for FUS–RNA systems, and T/T$_{\mathrm{c,FUS}}$=0.85 for PR$_{25}$–RNA mixtures. Error bars depict the computed standard deviation in the percentage contribution of electrostatic *vs.* non-electrostatic interactions. The polyU/FUS mass ratio was kept constant at a value of 0.096 for FUS-polyU simulations, and at a polyU/PR$_{25}$ mass ratio of 1.20 in PR$_{25}$-polyU simulations.

References

1. Joseph JA, Reinhardt A, Aguirre A, Chew PY, Russell KO, Espinosa JR, et al. Physics-driven coarse-grained model for biomolecular phase separation with near-quantitative accuracy. Nature Computational Science. 2021;1(11):732–743.

2. Debye P, Hückel E. De la theorie des electrolytes. I. abaissement du point de congelation et phenomenes associes. Physikalische Zeitschrift. 1923;24(9):185–206.

3. Wang X, Ramírez-Hinestrosa S, Dobnikar J, Frenkel D. The Lennard-Jones potential: when (not) to use it. Physical Chemistry Chemical Physics. 2020;22(19):10624–10633.

4. Espinosa JR, Garaizar A, Vega C, Frenkel D, Collepardo-Guevara R. Breakdown of the law of rectilinear diameter and related surprises in the liquid-vapor coexistence in systems of patchy particles. J Chem Phys. 2019;150:224510. doi:10.1063/1.5098551.

5. Espinosa JR, Joseph JA, Sanchez-Burgos I, Garaizar A, Frenkel D, Collepardo-Guevara R. Liquid network connectivity regulates the stability and composition of biomolecular condensates with many components. Proceedings of the National Academy of Sciences. 2020;.

6. Sanchez-Burgos I, Espinosa JR, Joseph JA, Collepardo-Guevara R. Valency and binding affinity variations can regulate the multilayered organization of protein condensates with many components. Biomolecules. 2021;11(2):278.

7. Sanchez-Burgos I, Joseph JA, Collepardo-Guevara R, Espinosa JR. Size conservation emerges spontaneously in biomolecular condensates formed by scaffolds and surfactant clients. bioRxiv. 2021;.

8. Jover J, Haslam AJ, Galindo A, Jackson G, Müller EA. Pseudo hard-sphere potential for use in continuous molecular-dynamics simulation of spherical and chain molecules. Journal of Chemical Physics. 2012;137(14). doi:10.1063/1.4754275.

9. Jones JE. On the determination of molecular fields.—I. From the variation of the viscosity of a gas with temperature. Proceedings of the Royal Society of London Series A, Containing Papers of a Mathematical and Physical Character. 1924;106(738):441–462.

10. Joseph JA, Espinosa JR, Sanchez-Burgos I, Garaizar A, Frenkel D, Collepardo-Guevara R. Thermodynamics and kinetics of phase separation of protein-RNA mixtures by a minimal model. Biophysical Journal. 2021;120(7):1219–1230.

11. Nosé S. A unified formulation of the constant temperature molecular dynamics methods. The Journal of Chemical Physics. 1984;81(1):511–519. doi:10.1063/1.447334.

12. Hoover WG. Canonical dynamics: Equilibrium phase-space distributions. Phys Rev A. 1985;31:1695–1697. doi:10.1103/PhysRevA.31.1695.

13. Plimpton S. Fast parallel algorithms for short-range molecular dynamics. Journal of Computational Physics. 1995;117(1):1–19. doi:10.1006/jcph.1995.1039.

14. Ladd AJC, Woodcock LV. Triple-point coexistence properties of the lennard-jones system. Chemical Physics Letters. 1977;51(1):155–159. doi:10.1016/0009-2614(77)85375-X.

15. García Fernández R, Abascal JLF, Vega C. The melting point of ice Ih for common water models calculated from direct coexistence of the solid-liquid interface. The Journal of Chemical Physics. 2006;124(14):144506. doi:10.1063/1.2183308.

16. Espinosa JR, Sanz E, Valeriani C, Vega C. On fluid-solid direct coexistence simulations: The pseudo-hard sphere model. Journal of Chemical Physics. 2013;139(14). doi:10.1063/1.4823499.

17. Rowlinson JS, Widom B. Molecular theory of capillarity. Courier Corporation; 2013.

18. Zollweg JA, Mulholland GW. On the law of the rectilinear diameter. The Journal of Chemical Physics. 1972;57(3):1021–1025.

19. Dignon GL, Zheng W, Kim YC, Best RB, Mittal J. Sequence determinants of protein phase behavior from a coarse-grained model. PLoS Computational Biology. 2018;14(1). doi:10.1371/journal.pcbi.1005941.

20. Krainer G, Welsh TJ, Joseph JA, Espinosa JR, Wittmann S, de Csilléry E, et al. Reentrant liquid condensate phase of proteins is stabilized by hydrophobic and non-ionic interactions. Nature Communications. 2021;12(1):1–14.
